# Supplementary material for: Task-oriented machine learning surrogates for tipping points of agent-based models
Source: Nat Commun. 2024 May 15;15:4117. doi: 10.1038/s41467-024-48024-7 (PMC11096392; doi:10.1038/s41467-024-48024-7)
Supplement: Supplementary file 1 — Supplementary Information [file 41467_2024_48024_MOESM1_ESM.pdf]

Supplementary Information: Task-Oriented Machine Learning Surrogates for Tipping Points of Agent-Based Models

Gianluca Fabiani[1,2], Nikolaos Evangelou[2], Tianqi Cui[2], Juan M. Bello-Rivas[3], Cristina P. Martin-Linares[4], Constantinos Siettos[5,*], and Ioannis G. Kevrekidis[2,3,6,*]

[1]Modelling Engineering Risk and Complexity, *Scuola Superiore Meridionale*, Naples 80138, Italy
[2]Dept. of Chemical and Biomolecular Engineering, *Johns Hopkins University*, Baltimore, MD 21218, USA
[3]Dept. of Applied Mathematics and Statistics, *Johns Hopkins University*, Baltimore, MD 21218, USA
[4]Dept. of Mechanical Engineering, *Johns Hopkins University*, Baltimore, MD 21218, USA
[5]Dipartimento di Matematica e Applicazioni "Renato Caccioppoli", *Università degli Studi di Napoli Federico II*, Naples 80126, Italy
[6]School of Medicine's Dept. of Urology, *Johns Hopkins University*, Baltimore, MD 21218, USA
[*]Corresponding authors, emails: `yannisk@jhu.edu`, `constantinos.siettos@ unina.it`

A Details on the financial market agent-based model

In order to obtain a concise description at the level of population dynamics, Omurtag and Sirovich derived a mesoscopic Fokker-Plank-type (FP) IPDE [1] (a continuity equation) for the agent probability density function $\rho(t,x)$, where the spatial variable corresponds to the preference state of the agents, i.e. $x \equiv X$. For the particular problem, at the limit of infinitely many agents and averaged along many possible trajectories, the continuity equation in terms of a probability flux $J(t,x)$ and a source $Q(t,x)$ reads:

$$\begin{aligned}\frac{\partial \rho(t,x)}{\partial t} &= -\frac{\partial J(t,x)}{\partial x} + Q(t,x),\\ \text{with } J(t,x) &= -\frac{1}{2}\sigma^2\frac{\partial \rho(t,x)}{\partial x} - \mu\rho(t,x),\end{aligned} \tag{1}$$

where, if we denote the fluxes at the boundaries $J(t,\pm 1) = J^{\pm}$, the source $Q(t,x)$ can be set to be $Q(t,x) = (J^+ + J^-)\delta(x)$ to compensate for the creation/disappearance of density at the boundaries.

The above equation can be written as:

$$\begin{aligned}\frac{\partial \rho(t,x)}{\partial t} = \frac{1}{2}\sigma^2(t)\frac{\partial^2 \rho(t,x)}{\partial x^2} + \frac{\partial(\mu(t,x)\rho(t,x))}{\partial x} + \\ + (J^+ + J^-)\delta(x).\end{aligned} \tag{2}$$

In the above, $\sigma^2(t)$ is the time-dependent diffusivity coefficient given by:

$$\sigma^2(t) = \nu^+(\epsilon^+)^2 + \nu^-(\epsilon^-)^2, \tag{3}$$

μ is the time-dependent, space-dependent drift coefficient, given by:

$$\mu(t,x) = \gamma x - \nu^+\epsilon^+ - \nu^-\epsilon^-. \tag{4}$$

$J^{\pm}$ denote the *inflow* and *outflow* through the boundaries that are restored/re-injected at the origin through a Dirac $\delta(x)$ in Eq. (2), in order to maintain agent conservation. Indeed, since ρ is a probability distribution, the equation has also to satisfy the normalization property:

$$\int_{-1}^{1} \rho(t,x)dx = 1, \quad \forall t; \tag{5}$$

it is convenient to consider homogeneous Dirichlet boundary conditions $\rho(\pm 1, t) = 0$ and the agents there instantaneously reset back to $X = 0$. In addition, the fluxes at

the boundaries $J(t, \pm 1) = J^{\pm}$ are given by:

$$J^{\pm}(t, x) = \mp \frac{1}{2}\sigma^2 \frac{\partial \rho(t, x)}{\partial x}\Big|_{x=\pm 1}, \quad (6)$$

reflecting the resetting process of agents that cross the boundaries.

Besides, in order to solve/integrate the Eq. (2), one has to find algebraic closures for the time-evolving diffusivity σ and drift μ coefficients. In [1], a mean field approximation of the buying/selling rates was proposed:

$$R^{\pm} = \pm \nu^{\pm} \int_{\pm 1 \mp \epsilon^{\pm}}^{\pm 1} \rho(x, t) dx. \quad (7)$$

Finally, based on Equation (in the main text):

$$\nu^{\pm}(t) = \nu^{\pm}_{ex} + gR^{\pm}(t), \quad (8)$$

and Eq. (7), one obtains:

$$\nu^{\pm} = \frac{\nu^{\pm}_{ex}}{1 - g \int_{\pm 1 \mp \epsilon^{\pm}}^{\pm 1} \rho(x, t) dx}, \quad (9)$$

from which one can retrieve at each time instance t, the coefficients $\mu(t)$ and $\sigma^2(t)$ of the FP model, as defined in Eqs. (3)-(4).

The designation "Fokker-Planck" notwithstanding, it is important to restate that the above approximation is an integro-differential equation with space-dependent coefficients.

B Details on the Epidemic ABM

Compartmental models serve as structured population models, where the population is categorized based on their roles in the epidemiological process. We consider a stochastic version of a susceptible-infected-recovered-susceptible (SIRS) in discrete time. The rules that drive the model are presented in the main text. Here we give more details about the structure of the network on which the dynamics evolves. This network is constructed in a straightforward manner: within a population of N nodes, it is assumed that each node can potentially be connected to any of the other $N-1$ nodes with a probability p. This implies that a node has an equal probability, denoted as p, of forming a connection with every other node in the network. Therefore, the degree distribution of Erdös-Rényi network follows the binomial law:

$$P(k) = \binom{N-1}{k} p^k (1-p)^{N-1-k}, \quad (10)$$

where k denote a degree value. The mean degree of the network is $\mathbb{E}(k) = \bar{k} = pN$. This distribution for $p = \frac{1}{2}$ is exactly symmetric, while for other values of $p < 1/2$ is almost symmetric but with a "long tail", i.e. there is a very low probability for the occurrence of degrees $k > 2pN$. In our computation we selected $p = 0.0008$ and $N = 10,000$ which gives $\bar{k} = 8$. In particular along all computation we have used a predetermined Erdös-Rényi network, with the maximum degree of a node being $k_{max} = 21$.

C Equation-Free approach

The Equation-Free (EF) framework, proposed in [2], operates on the key assumption that for a given microscopic simulator there exists a fundamental coarse description: as the distributions evolve, higher-order moments quickly become dependent on lower-order ones, ultimately converging towards a slow invariant manifold. This concept embodies the singularly perturbed system paradigm, where the interconnected nonlinear ordinary differential equations governing the moments of the agent distribution rapidly approach a low-dimensional slow manifold. The main tool employed in the EF approach are the so-called *coarse time-steppers*. Coarse time-steppers establish a link between microscopic simulators, such as ABMs and traditional continuum numerical algorithms. Such methods encompass a series of essential stages, as outlined below:

- Assume we start from a coarse-scale initial condition, corresponding to the appropriate coarse-scale variables $\boldsymbol{x}(t)$ (e.g., diffusion map coordinates) of the evolving ensemble of agents at time t;

- Map the macroscopic description, $\boldsymbol{x}(t)$, through a *lifting operator*, $\mathcal{L}$, to an ensemble of consistent microscopic realizations:

$$X(t) = \mathcal{L}(\boldsymbol{x}(t)); \quad (11)$$

- For an (appropriately chosen) short macroscopic time ΔT, evolve these realizations using the black-box ABM simulator to obtain the value(s):

$$X(t + \Delta T) = \Phi_{\Delta T}(X(t)) \equiv \Phi_{\Delta T}(\mathcal{L}\boldsymbol{x}(t)); \quad (12)$$

- Map the ensemble of agents back to the macroscopic description through the *restriction operator* $\mathcal{M}$:

$$\boldsymbol{x}(t + \Delta T) = \mathcal{M}X(t + \Delta T). \quad (13)$$

The entire procedure, i.e., the coarse time-stepper, can be thought of as a "black box":

$$\boldsymbol{x}(t+\Delta T) = \phi_{\Delta T}[\boldsymbol{x}(t)] \equiv \mathcal{M}\Phi_{\Delta T}(\mathcal{L}[\boldsymbol{x}(t)]). \tag{14}$$

Such an approach, allows one to accelerate simulations and also to perform bifurcation analysis [2]. To find a stationary state $\boldsymbol{x}$ of eq.(14), if there exists, we seek a solution of the following equation:

$$\boldsymbol{F}(\boldsymbol{x}) = \boldsymbol{x} - \phi_{\Delta T}[\boldsymbol{x}] = 0, \tag{15}$$

wrapping around it the Newton-GMRES method [3].

D Machine Learning algorithms used

D.1 Diffusion Maps: a Dimensionality Reduction Approach

Diffusion Maps (DMAPs), proposed by Coifman and Lafon [4, 5], is a manifold learning technique capable of discovering linear and non-linear patterns in high-dimensional data: an intrinsic embedding of the low-dimensional manifold on which the data (are assumed to) lie.

The algorithm, using a random walk on the available data points (each considered as the node of a graph), discovers the underlying structure of the data geometry by approximating the Laplace-Beltrami operator on the data manifold. In this graph the edges between nodes (data points) represent the probability of transitioning from one data point to another. Starting with the data matrix $\mathbf{X} \in \mathbb{R}^{m \times d}$, where m is the number of data points, and d is the dimension of each data point $\boldsymbol{x}_i$, the DMAPs algorithm constructs an affinity matrix (kernel matrix) W: the entries of W are computed as:

$$w_{ij} = \exp\left(-\frac{||\boldsymbol{x}_i - \boldsymbol{x}_j||^2}{2\epsilon}\right). \tag{16}$$

where ϵ is a positive scale parameter. The metric $||\cdot||$ we consider here is the ℓ^2 norm.

To make the kernel matrix invariant to the sampling density, and to ensure numerical approximation of the Laplace Beltrami operator the normalization

$$\tilde{W} = P^{-1}WP^{-1}, P_{ii} = \sum_{j=1}^{m} W_{ij} \tag{17}$$

is applied.

Then the matrix $D \in \mathbb{R}^{m \times m}$ is constructed as $D_{ii} = \sum_{j=1}^{m} \tilde{W}_{ij}$ and the second normalization

$$A = D^{-1}\tilde{W} \tag{18}$$

is applied to obtain the row-stochastic matrix A.

We then compute the eigendecomposition of A,

$$A\psi_i = \lambda_i \psi_i, \tag{19}$$

with the eigenvectors ψ_i sorted based on the eigenvalues λ_i. Proper selection of the eigenvectors that span independent eigendirections (termed non-harmonics) is necessary. The selection of the non-harmonic eigenvectors is here obtained by applying the local linear regression algorithm proposed by Dsilva et al. [6] on the computed eigenvectors. This linear regression algorithm by fitting eigenvectors ψ_i (where $i > 1$) as local linear functions of previous eigenvectors can detect the non-harmonic eigenvectors. A normalized leave-one-out error, denoted as r_k, is used for this selection and quantifies gradually which eigenvectors are independent (non-harmonic) and which are not (harmonic). If the number of non-harmonic eigenvectors is smaller than d the dimensionality reduction is achieved.

D.2 Feedforward Neural Networks

Feedforward Neural Networks (FNN) are a class of powerful machine learning tools, characterized by a layered structure of interconnected computing units (neurons), that are nowadays widely used for supervised learning tasks, such as regression, classification, forecasting and model identification. The great popularity of FNN is due to their capability to approximate any (piecewise) continuous (multivariate) function, to any desired accuracy, as it is stated in the celebrated universal approximation theorem [7, 8]. This implies that any failure of a network must arise from an inadequate choice/calibration of weights and biases or an insufficient number of hidden nodes.

Let us consider a FNN with a N_0-dimensional input $\boldsymbol{y}^0$, with L hidden-layers composed by N_l neurons. The output $y_j^{(l)}$ of the j-th neuron ($j = 1, \dots, N_l$) in the l-th layer ($l = 1, \dots, L$) consists of an evaluation of the so-called activation function $\psi : \mathbb{R} \mapsto \mathbb{R}$ of a linear combination of neurons' outputs of the previous layer:

$$y_j^{(l)} = \sum_{i=1}^{N_l} \psi(w_{ji}^{(l)} y_i^{(l-1)} + b_i^{(l)}) \tag{20}$$

where the weights $w_{ji}^{(l)}$ are the weights of the connection between neurons i and j belonging to two consecutive layers, and $b_i^{(l)}$s are the so-called biases. If we denote by $\Phi^l : \boldsymbol{y}^{(l-1)} \in \mathbb{R}^{N_{l-1}} \mapsto \boldsymbol{y}^l \in \mathbb{R}^{N_l}$ the map between the $(l-1)$-st layer to the l-th layer, then we can express the output $\boldsymbol{y}^{L+1}$ of the network as the composition of all the layer maps:

$$\boldsymbol{y}^{(L+1)} = \Phi^{(L+1)} \circ \cdots \circ \Phi^1(\boldsymbol{y}^{(0)}). \tag{21}$$

D.2.1 Training of the FNN

For supervised learning tasks, the goal is to find an *optimal* (most of the times is just sub-optimal) configuration of weights and biases that minimize a loss/cost function, usually defined as the Mean-Squared error between the current output of the network and the desired output $\boldsymbol{d} = (d_1, \ldots, d_k, \ldots, d_M)$ along a collection of M data samples $\boldsymbol{y}_1^{(0)}, \ldots, \boldsymbol{y}_k^{(0)}, \ldots, \boldsymbol{y}_M^{(0)}$ that constitute the so-called training set:

$$E = \sum_{k=1}^{M} ||\boldsymbol{d}_k - \boldsymbol{y}_k^{(L+1)}||_2^2. \tag{22}$$

For our computations we used the Bayesian regularized back-propagation updating the weight values using the Levenberg-Marquardt algorithm [9] as implemented in the Deep Learning toolbox of `Matlab` 2021a. For learning the IPDE we used a FNN with two hidden layers, both with 16 Neurons employing a hyperbolic tangent activation function. Levenberg-Marquardt emulates a second-order Newton-like scheme by computing an approximation of the Hessian matrix $H_{\boldsymbol{p}}$ of the loss function as:

$$H_{\boldsymbol{p}} \approx J_{\boldsymbol{p}}^T J_{\boldsymbol{p}}, \tag{23}$$

where $J_{\boldsymbol{p}}$ is the Jacobian matrix of the network errors with respect to weights and biases. Thus, the vector $\boldsymbol{p}$ collecting all trainable parameters is iteratively adjusted as follow:

$$\boldsymbol{p} \leftarrow \boldsymbol{p} - (J_{\boldsymbol{p}}^T J_{\boldsymbol{p}} + \mu I)^{-1} J_{\boldsymbol{p}}^T \boldsymbol{e}, \tag{24}$$

where $\boldsymbol{e}$ is a vector of network errors.

D.3 Random Projection Neural Networks

Random Projection Neural Networks (RPNNs) are a family of Artificial neural networks including Random Vector Functional Links (RVFLs) [10], Random Fourier Features (RFF) [11], Reservoir Computing [12, 13] and Extreme Learning Machines (ELMs) [14]. For a brief review of RPNNs see our paper [15]. Here, for simplicity we consider a feedforward RPNN, with one single hidden layer, one output layer with linear activation function and zero output bias $b_i^{(2)} = 0$, that can be written as:

$$y^{(2)} = \sum_{i=1}^{N} w_i^{(2)} \psi(\boldsymbol{w}_i^{(1)} \boldsymbol{y}^{(0)} + b_i^{(1)}) \tag{25}$$

and we consider a training set of M points $\boldsymbol{y}_1^{(0)}, \ldots, \boldsymbol{y}_k^{(0)}, \ldots, \boldsymbol{y}_M^{(0)}$ with desired outputs $\boldsymbol{d} = (d_1, \ldots, d_k, \ldots, d_M)$. Randomly fixing $\boldsymbol{w}_i^1$ and $b_i^{(1)}$, we can rewrite (25) as a linear system in the unknowns $\boldsymbol{w}^{(2)}$:

$$\boldsymbol{d} = \boldsymbol{w}^{(2)} \Phi \tag{26}$$

where $\Phi \in \mathbb{R}^{N \times M}$ is the matrix with elements:

$$\Phi_{ik} = \psi(\boldsymbol{w}_i^{(1)} \boldsymbol{y}_k^{(0)} + b_i^{(1)}). \tag{27}$$

Therefore, one can find the output weights as a linear least square problem, here using the `pinv` of `Matlab`, employing the Moore-Penrose pseudo-inverse (with a regularizing tolerance 1e−6).

Random Fourier Features (RFF) [11] is a particularly interesting and straightforward RPNN approach, that uses the cosine as the activation function of the network, i.e., $\psi(\cdot) = cos(\cdot)$. This choice leads to a random Fourier basis expansion approximation. As proposed in [11], we sampled the weights that connect the input and the hidden layer from the following distribution

$$p(w) = 2\pi^{(-\frac{D}{2})} \exp\left(-\frac{||w||_2^2}{2}\right), \tag{28}$$

where D is the dimension of the input space. For the choice of the biases we used an uniform distribution in the interval $[0, 2\pi]$. For learning the IPDE we used a a single hidden layer RFF with 350 neurons.

E Details on Learning the Integro-Partial Differential Equation for the finance ABM

E.1 Training and test data sets

ABM simulations were performed using $N = 50,000$ agents, with $\nu_{ex}^{+} = \nu_{ex}^{-} = 20$, $\gamma = 1$, $\epsilon^{-} = -0.072$,

$\epsilon^+ = 0.075$. The mimetic strength g is our bifurcation parameter. For the data set, we selected 41 equally-spaced points in the range $g \in [30, 50]$ and for each of the values of g, we randomly generated 1000 different initial profiles ρ_0, as Gaussian distributions $\rho_0 \sim \mathcal{N}(\tilde{m}_0, \tilde{s}_0^2)$ with varying mean $\tilde{m}_0$ and variance $\tilde{s}_0^2$. The initial $\tilde{m}_0$ and $\tilde{s}_0$ were uniformly randomly sampled as $\tilde{m}_0 \sim \mathcal{U}([-0.3, 0.3])$, $\tilde{s}_0 \sim \mathcal{U}([0.3, 0.5])$. The initial state of the agents is sampled from the initial distribution ρ_0, creating a consistent microscopic realization. At each time step, as the agents dynamically evolve, to estimate the corresponding coarse-grained density profile, we used 81 equally-spaced bins, with equispaced centers $x_i \in [-1, 1]$. Since we are dealing with a stochastic model, in order to generate smooth enough profiles for the spatial derivatives, for each fixed initial condition, we ran 100 random stochastic realizations, and we averaged along the generated copies of the density field. Then to further smooth out the computed densities, we applied a weighted moving average smoothing ρ_i as $\rho_i^\star = \frac{2\rho_i + \rho_{i-1} + \rho_{i+1}}{4}$. ($i$ denotes spatial mesh points).

The ABM simulations were run for a time interval $t \in [0, 15]$ and we collected points with a time step of $\Delta t = 0.25$. When the mean value $\bar{X}$ crossed, ± 0.4 we stopped the simulations, because the pdf profile blows up, very fast after that. Furthermore, we also ignored the first two time points, to exclude the initial "healing" transients due to the way we initialize (see the discussion for such healing periods in [2]). We thus end up with a data set consisting of 40 (values of g) $\times 100$ (initial conditions) $\times 58$ (maximum time points ignoring the first 2 steps of the transient) $\times 81$ (space points) $\approx 15 \times 10^6$ data points. Since the amount of data is practically too large, for the training set we have randomly downsampled to 10^6 data and used the remaining data as our test set.

E.2 Feature selection for the mesoscopic IPDE model

For dealing with the "curse of dimensionality" in training the FNN, learning our IPDE model, we used ARD as implemented in `Matlab` by the function `fitrgp` for feature selection. Here, we *a priori* selected as candidate features, the space x *per se*, the field ρ, the first ρ_x, second ρ_{xx} and third ρ_{xxx} spatial derivatives estimated with central finite differences, as well as $I^\pm$ defined as the integrals of ρ in a small region close to the boundaries:

$$I^\pm(t) = \pm \int_{\pm 1 \mp \epsilon}^{\pm 1} \rho(x, t) dx, \tag{29}$$

where $\epsilon = 0.05$ corresponds to the size of the last two bins of the grid. We note that the latter two candidate mesoscopic variables $I^\pm$ as defined above are related to the buying and selling rates (see Eq.7), yet they do not depend on the frequencies $\nu^\pm$ as the true buying and selling rates do. These "internal"/hidden variables $\nu^\pm$ are considered unknown. We also consider unknown the "quantum" jump sizes $\epsilon^\pm$. The target variable to learn is the time derivative, at each collocation point x, estimated with forward FD as:

$$\rho_t(x, t) = \frac{\partial \rho(x, t)}{\partial t} = \frac{\rho(x, t + dt) - \rho(x, t)}{dt}. \tag{30}$$

The effective relevance weights $W_r(\cdot)$ of the features, as obtained by using ARD, are $W_r(\rho) = 0.25$, $W_r(\rho_x) = 0.22$, $W_r(\rho_{xx}) = 0.14$, $W_r(\rho_{xxx}) = 0.04$, $W_r(I^+) = 0.18$, $W_r(I^-) = 0.12$, $W_r(x) = 0.27$. As can be noted, the third derivative is the least important feature, and we thus decided to disregard it; the most important feature is the space x highlighting that the IPDE is not translational invariant. The dependency on x implicitly captures the location of the source term Q representing the resetting of the state of the agents at the origin as in the FP IPDE Eq. (2). Note that the integral features $I^\pm$ are also important. This is in line with the theoretical results regarding the FP IPDE Eq. (2).

F Details on Learning the Stochastic Differential Equations for the financial ABM

F.1 Data collection and preprocessing

In this section we describe how we collected data, specifically targeted to the neighborhood of the tipping point, for the purpose of learning a parametric mean-field SDE. ABM simulations were performed using $N = 50{,}000$ agents, with $\nu^\pm = 20$, $\epsilon^+ = 0.075$, $\epsilon^- = -0.072$, $\gamma = 1$. We selected 11 equally-spaced values of the mimetic strength g, which is used as bifurcation parameter, in the range $g \in [42, 47]$. We gathered at each time stamp the state of every agent (X_i), as well as the overall buying/selling rates (R^+ and R^-). For convenience, we use

the vector $\boldsymbol{s} = (X_1, X_2, \cdots, X_n, R^+, R^-)^T$, and denote the mean preference value of the agent state as:

$$\bar{X} = \frac{1}{n}\sum_{i=1}^{n} X_i.$$

To select initial conditions that populate the state space (in terms of $\bar{X}$, across multiple values of the parameter g) the following protocol was used: We start by running one trajectory of the full ABM for $g = 47$, which ultimately leads to an explosion. This trajectory was initialized by sampling the agents from a triangular distribution $p(X)$, as implemented in python, with lower limit -1, upper limit 1, and mode -0.6, corresponding to a value $\bar{X} \approx -0.2$. This trajectory was stopped using the termination condition $\bar{X} \leq 0.4$, indicative of incipient explosion. We selected 25 distinct instances of the state of agents along the stochastic trajectory, corresponding to 25 different values of $\bar{X}$ in the range $[-0.02, 0.32]$. Then, for each value of the parameter g, we simulated a total of 50 new stochastic trajectories, two for each distinct initial condition.

As previously done in [16], in order to find the data-based one-dimensional coarse observable ψ_1, the DMAPs algorithm is carried out on 39 intermediate coarse variables. Thus, we set up 37 percentile points $p_1, p_2, \cdots, p_{37}$ referring to our discretization of the cumulative distribution function (cdf) of the agents' preference state. For each p_i, we computed its quantile function value $Q(p_i)$, where the quantile function $Q(\cdot)$ is defined as the inverse function of (cdf) F_X of the random variable X. The first 19 percentile values $p_1, p_2, \cdots, p_{18}, p_{19}$ are set as 0.0005, 0.001, 0.002, 0.003, 0.004, 0.005, 0.0075, 0.01, 0.02, 0.03, 0.04, 0.05, 0.075, 0.1, 0.15, 0.2, 0.3, 0.4, 0.5. The last 18 probability values are set to be symmetric with the first 18, i.e. $p_i = 1 - p_{38-i}, \quad \forall i = 20, 21, \cdots, 37$; The remaining two coarse variable are the overall buying and selling rates. Therefore, the full state $\boldsymbol{s}$ has a coarse 39-dimensional representation $\boldsymbol{s'} = (Q(p_1), Q(p_2), \cdots, Q(p_{37}), R^+, R^-)^T$.

F.2 Statistical mechanics-based computation of escape times

The second approach for estimating escape times involves numerical computation of the escape times based on the known closed-form expression for the one-dimensional SDE:

$$dx_t = \mu(x_t; \varepsilon)dt + \sigma(x_t; \varepsilon)dW_t. \tag{31}$$

Here we report the expressions for the constant diffusivity case, since the identified diffusivities in our case appear not to be state (or parameter) dependent, see Section F. The effective potential U_e for a one-dimensional SDE with constant diffusivity is given by:

$$U_e(x) = \int_{x_0}^{x} -\frac{\mu(x)}{\sigma^2/2}dx, \tag{32}$$

where x_0 is an arbitrarily selected reference point in which the potential is taken to be zero, $\mu(x)$ is the drift, and σ is the diffusivity. Equation (32) is derived from the steady-state solution of the Fokker-Planck equation, see the Section C in [17].

It has been pointed out in [18] that, there is a closed-form expression of escape times for one-dimensional systems with constant diffusivity (33).

$$dx_t = -\nabla U_e(x_t)dt + \sqrt{\frac{2}{\beta}}dW_t, x_0 \in (a, b), \tag{33}$$

where $U_e(x_t)$ is the effective potential of the system, (a, b) are the left and right boundaries of the interval of interest, and β is the inverse temperature, inspired from thermodynamics. Compared with (31), we can transform our learned SDE form into (33) by setting

$$\mu(x_t) = -\nabla U_e(x_t) = -\frac{dU_e(x_t)}{dx_t}; \sigma = \sqrt{\frac{2}{\beta}}. \tag{34}$$

Notice that one can also consider the drift term $\mu(x_t)$ as the force term in an energy field. The mean escape time for systems like (33) can be written as a univariate integral:

$$\langle \tau \rangle = \int_a^b \rho(x; x_0)dx, \tag{35}$$

where $\rho(x; x_0)$ is the occupation density of particles at $x = x_0$ which solves the boundary value problem (see Section F.2),

$$\begin{cases} -\frac{d}{dx}\left(\beta^{-1}\rho'(x) + \rho(x)U_e'(x)\right) = \delta(x - x_0), & \text{in } (a, b), \\ \rho(a) = \rho(b) = 0. \end{cases}$$

The occupation density ρ can be computed as the solution of the above boundary value problem:

$$\rho(x; x_0) = \beta \int_a^x (G(x_0) - H(z - x_0))e^{\beta(U_e(z) - U_e(x))}dz, \tag{36}$$

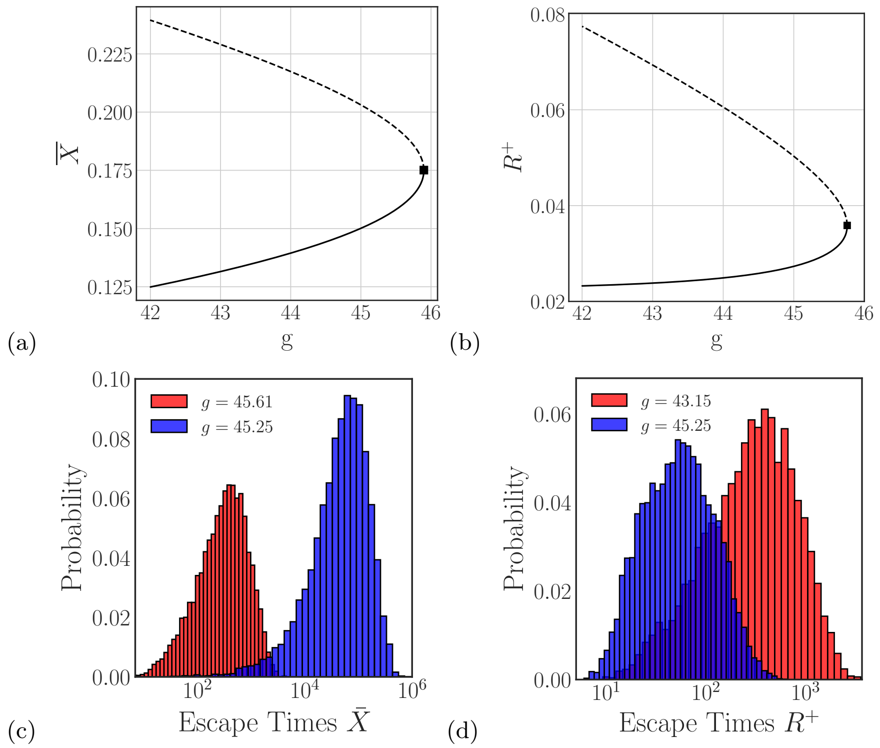

Supplementary Figure 1: Additional results for two alternative SDEs surrogates. (a)-(b) The constructed bifurcation diagrams are based on the drift component of the identified effective SDEs in terms of (a) $\bar{X}$, and of (b) R^+. The bifurcation point is marked with a black square. (c)-(d) Histograms of escape times obtained with simulations of 10,000 stochastic trajectories for the SDE models trained on (c) $\bar{X}$, (d) R^+.

where $H(\cdot)$ is the Heaviside step function and $G(\cdot)$ is defined as

$$G(x_0) = \frac{\int_{x_0}^{b} e^{\beta U_e(\eta)} d\eta}{\int_{a}^{b} e^{\beta U_e(\eta)} d\eta}. \tag{37}$$

The above analysis does not depend on the choice of potential reference point. Consider another representation of the potential $U_e'(\cdot)$ that actually has

$$U_e'(\eta) - U_e(\eta) = C \tag{38}$$

for $\forall \eta \in \mathbb{R}$, where C is a constant. One can substitute (38) in (37) to get

$$\begin{aligned} G'(x_0) &= \frac{\int_{x_0}^{b} e^{\beta U_e'(\eta)} d\eta}{\int_{a}^{b} e^{\beta U_e'(\eta)} d\eta} = \frac{\int_{x_0}^{b} e^{\beta (U_e(\eta)+C)} d\eta}{\int_{a}^{b} e^{\beta (U_e(\eta)+C)} d\eta} = \\ &= \frac{e^{\beta C} \int_{x_0}^{b} e^{\beta U_e(\eta)} d\eta}{e^{\beta C} \int_{a}^{b} e^{\beta U_e'(\eta)} d\eta} = G(x_0); \end{aligned} \tag{39}$$

this implies that the function $G(\cdot)$ is independent of the reference point. As the exponential term in (36) is a function of the difference of potential values, the choice of the reference point will not affect the value of the occupation density $\rho(\cdot)$, nor the values of escape times $\langle \tau \rangle$. In practice, the integration can be performed numerically using a quadrature scheme. However, quadrature schemes are susceptible to numerical errors under some circumstances. For example, if the diffusivity is small, $\sigma \ll 1$ (and thus β is large), this leads to exponential values that might be numerically intractable and impede the utilization of the method.

To address this issue, we transform the SDE (31) by $x \mapsto y(x) = x/\sigma$ and apply Itô's lemma to obtain the SDE,

$$dy_t = \sigma^{-1} \mu(\sigma y_t; g) dt + dW_t. \tag{40}$$

where the diffusivity in this case is one.

This transformation allows us to compute the escape times in terms of the transformed variables y_t and using $\beta = 2$, which alleviates the numerical issues. The escape times for the transformed variable y_t and the original variables are the same. This allows us to transform

the SDEs identified from the trained neural networks to compute the escape times using (35). It is worth mentioning that the applied transformation, even though it circumvents the numerical issues arising because of the diffusivity in cases where the potential is large (and therefore the exponent of the potential is also large), would still be susceptible to numerical issues in other regimes.

F.3 Additional Results: Two physical based alternative mean-field SDEs

In this section, we illustrate results obtained for two alternative one-dimensional mean-field SDE surrogates, using as their effective state variable $\bar{X}$ and R^+, respectively; these were omitted in the main text for brevity.

The constructed bifurcation diagrams from the identified deterministic drift of the two SDE surrogates trained on $\bar{X}$ and R^+ are shown in Supplementary Figure 1a-1b. The bifurcation point, in both cases, occurs for $g^* \approx 45.5$. Indeed, we obtained a tipping point at $g^* = 45.90$ with a corresponding $\bar{X}^* = 0.1751$ for the SDE trained on $\bar{X}$; and at $g^* = 45.76$, with a corresponding $R^{+*} = 0.003586$ for the SDE trained on R^+. The identified bifurcation point (our tipping point) for all three identified SDE models is consistent with the value reported in Liu P. et al [3, 16, 19].

For each of the identified SDEs, in terms of $\bar{X}$ and R^+ respectively, we estimated escape time distribution by computing $10,000$ stochastic simulations. Histograms of the obtained escape times for $\bar{X}$ and R^+ are shown in Supplementary Figure 1c-1d, respectively. Means of escape times distribution and their standard deviations for the identified SDE in terms of $\bar{X}$ and R^+ here. We computed for the SDE on $\bar{X}$ a mean of 75627.73 and standard deviation 75198.20, for $g = 45.25$. While for $g = 45.61$ we got mean 468.06 and standard deviation of 456.71. For the SDE on R^+ at $g = 43.25$ we got mean 73.61 and standard deviation 63.95. While at $g = 43.15$ we got mean 436.59 and standard deviation 420.82. Similarly to the computations reported in the main text, for the SDE models in $\bar{X}$ and R^+ we estimated the mean escape times for $g = 45.25$; We also found parameter values of g that provide comparable mean escape time with that of the full ABM for $g = 45.25$.

The estimated escape times of the identified SDE in $\bar{X}$ for $g = 45.25$ is much larger than any other model (including the ABM). This might suggest that this surrogate model might be unreliable. The surrogate SDE model constructed in R^+ has an escape time similar to the model trained on ψ_1.

F.4 Additional Result: Escape Times by quadrature

In this Section we report results of the alternative approach discussed in Section F.2 to compute escape times for one-dimensional systems. This computation of mean escape times involves quadrature integration for the three different SDE surrogates in $\bar{X}, R^+, \psi_1$ coordinates, respectively. The transformation is discussed in Section F.2. The estimated mean escape times for $g = 45.25$ are reported here. For the SDE trained on ψ_1 we got mean 22.23. For the SDE trained on $\bar{X}$ the quadrature algorithm did not converge. For the SDE trained on R^+ we got mean 39.64.

As it can be seen these results, the estimated escape times for the SDEs on the R^+ and ψ_1 are comparable to the ones obtained with the Monte-Carlo simulations (reported in the main text in the Paragraph about Rare-event analysis of catastrophic shifts). However, the escape times obtained with the SDE trained on $\bar{X}$ did not converge because of overflow errors. The latter is due to numerical issues that arise in the quadrature involving the computation of the exponential of the potential in Eq. (37).

Model	z	β	a	b
$\sigma^{-1}\bar{X}$	43.89	2	10.00	62.71
$\sigma^{-1}R^+$	4.34	2	0.00	8.65
$\sigma^{-1}\psi_1$	41.87	2	36.91	48.00

Supplementary Table 1: The parameter values used for the escape time computations with the quadrature method across the three transformed models.

We now discuss in more detail how the parameters α, β and z, specifying the limits of the integrals in equation 37, were selected for the quadrature integration:

1. The parameter z indicates the point where the process is initiated. In our case this was selected to be the stable steady state identified by the drift network for a fixed value of the parameter $g = 45.25$. We set the value of the potential for $U_e(\cdot)$ to be equal to zero.

2. The parameter β is estimated based on the diffusivity σ of the SDE. Assuming that $\sigma = \sqrt{2\beta^{-1}}$ and thus $\beta = \frac{2}{\sigma^2}$. Because β is being raised to an exponent for the quadrature computations, when σ is not close to one, numerical overflow can arise. This is the reason we decided to perform Itô's transformation to the SDE mentioned in Section F.2 and shown in Eq. (40).

3. The parameters a and b denote the limits of the integral. For the SDEs trained on R^+ and $\bar{X}$ we set a as the point beyond which the probability of observing the trajectory is negligible. This assumes that the potential energy becomes too large beyond this point. We set b as the value of the state variable (R^+ or $\bar{X}$) at which the trajectory has already surpassed the unstable steady state. The role of a and b for the SDE trained on ψ_1 is reversed since the potential is flipped.

In Supplementary Table 1 we report the parameters z, β, a and b for the three SDE models. Please note that for all those cases the parameter β was set to $\beta = 2$ because of the applied Itô's transformation.

F.5 Details on Neural Network Architectures for learning SDEs

In this Section, we provide a short description of the four neural networks trained on the DMAPs coordinate ψ_1, the mean preference state $\bar{X}$, the buying rate R^+ and ϕ_1 to identify four different one-dimensional mean-field SDEs. For all four networks, we used 5 hidden layers with 32 neurons for each layer. The activation functions for the *drift* neural network were selected as `tanh` and for the diffusivity as `softplus`. The data were split 90|10 into training|validation subset.

G Details on the epidemic ABM simulations

G.1 Data collection for learning the ML mean-field-level SIR

ABM simulations were performed using $N = 10,000$ agents on an Erdös Rényi distributed network (see Section B).

The probability λ that susceptible individuals become infected is our bifurcation parameter. For the data set, we selected 51 equally spaced points in the range $\lambda \in (0, 0.5]$. For each of the values of λ, we created a grid of 146 points in the triangle with corners $[(0,0),(0,1),(1,0)]$. The grid points are concentrated close to the edges $[(0,0)-(0,1)]$ and $[(0,0)-(1,0)]$ and also close to the effective unstable steady-states, obtained by the EF approach in [20]. These values correspond to the initial values at time $t = 0$ of the densities $[S_0]$ and $[I_0]$ that are constrained by $[S]+[I] \leq 1$. These initial states are then lifted into the agents' space, i.e. randomly picking a fraction of $[S]$ nodes and a fraction of $[I]$ nodes in the network and assigning the corresponding state S and I. The remaining nodes are then set as R. At each time step t of the ABM, we collect the densities $[S](t)$ and $[I](t)$. Since we are dealing with a stochastic model, for each initial condition, we ran 20 stochastic simulations of the ABM, and we averaged along the generated copies (realizations) of the density variables.

The ABM simulations were performed for a time interval $t \in [0, 12]$ with a constant time step of $\Delta t = 1$ corresponding to 1 day in unit of time. We ignored the first two time points, to avoid the initial "healing" transients. We thus collected a data set consisting 51 (values of λ) $\times 146$ (initial conditions) $\times 10$ (time points) $\times 20$ (copies) $\approx 1,500,000$ data points. The estimation of the time derivative for learning the system of ML mean-field-level ODEs, was made with a three-point centered Finite Difference stencil.

G.2 Data collection for learning the ML SDE surrogate targeted in the neighborhood of the tipping point

We have in this case, focused our attention on a smaller region in parameter space, targeted to the tipping point, where the dynamics is effectively one-dimensional. In particular we have selected 21 parameter values in $\lambda \in [0.2, 0.4]$ and for each a fixed grid of 63 initial conditions in the box $([S_0], [I_0]) \in [0.05, 0.3] \times [0.25, 0.7]$. We have also run the ABM for 20 time instants, collecting 5 different stochastic realization (we do not average in this case, as we are interested in learning also the stochastic nature of the dynamics). We ignored the first five time points, to avoid the initial "healing" transients and also to keep only the long-term dynamics that live on the one-dimensional manifold. Therefore we obtained a data set consisting of 21 (values of λ) $\times 63$ (initial conditions) $\times 15$ (time points) $\times 5$ (different realizations) $\approx 100,000$ data

points. However for the ML SDE surrogate, the data were randomly subsampled to just $25,000$ data points.

Supplementary References

[1] Omurtag, A. & Sirovich, L. Modeling a large population of traders: Mimesis and stability. *Journal of Economic Behavior and Organization* **61**, 562–576 (2006). DOI 10.1016/j.jebo.2004.07.016.

[2] Kevrekidis, I. G. *et al.* Equation-free, coarse-grained multiscale computation: Enabling microscopic simulators to perform system-level analysis. *Communications in Mathematical Sciences* **1**, 715–762 (2003). DOI 10.4310/cms.2003.v1.n4.a5.

[3] Siettos, C., Gear, C. W. & Kevrekidis, I. G. An equation-free approach to agent-based computation: Bifurcation analysis and control of stationary states. *EPL (Europhysics Letters)* **99**, 48007 (2012). DOI 10.1209/0295-5075/99/48007.

[4] Coifman, R. R. *et al.* Geometric diffusions as a tool for harmonic analysis and structure definition of data: Diffusion maps. *Proceedings of the National Academy of Sciences* **102**, 7426–7431 (2005). DOI 10.1073/pnas.0500334102.

[5] Coifman, R. R., Kevrekidis, I. G., Lafon, S., Maggioni, M. & Nadler, B. Diffusion maps, reduction coordinates, and low dimensional representation of stochastic systems. *Multiscale Modeling & Simulation* **7**, 842–864 (2008). DOI 10.1137/070696325.

[6] Dsilva, C. J., Talmon, R., Coifman, R. R. & Kevrekidis, I. G. Parsimonious representation of nonlinear dynamical systems through manifold learning: A chemotaxis case study. *Applied and Computational Harmonic Analysis* **44**, 759–773 (2018). DOI 10.1016/j.acha.2015.06.008.

[7] Cybenko, G. Approximation by superpositions of a sigmoidal function. *Mathematics of Control, Signals and Systems* **2**, 303–314 (1989). DOI 10.1007/BF02551274.

[8] Hornik, K., Stinchcombe, M. & White, H. Multilayer feedforward networks are universal approximators. *Neural Networks* **2**, 359–366 (1989). DOI 10.1016/0893-6080(89)90020-8.

[9] Hagan, M. T. & Menhaj, M. B. Training feedforward networks with the marquardt algorithm. *IEEE Transactions on Neural Networks* **5**, 989–993 (1994). DOI 10.1109/72.329697.

[10] Pao, Y.-H. & Takefuji, Y. Functional-link net computing: theory, system architecture, and functionalities. *Computer* **25**, 76–79 (1992). DOI 10.1109/2.144401.

[11] Rahimi, A. & Recht, B. Random features for large-scale kernel machines. *Advances in neural information processing systems* **20** (2007). URL https://proceedings.neurips.cc/paper_files/paper/2007/file/013a006f03dbc5392effeb8f18fda755-Paper.pdf.

[12] Jaeger, H. The "echo state" approach to analysing and training recurrent neural networks-with an erratum note. *Bonn, Germany: German National Research Center for Information Technology GMD Technical Report* **148**, 13 (2001).

[13] Gauthier, D. J., Bollt, E., Griffith, A. & Barbosa, W. A. Next generation reservoir computing. *Nature communications* **12**, 1–8 (2021). DOI 10.1038/s41467-021-25801-2.

[14] Huang, G.-B., Zhu, Q.-Y. & Siew, C.-K. Extreme learning machine: theory and applications. *Neurocomputing* **70**, 489–501 (2006). DOI 10.1016/j.neucom.2005.12.126.

[15] Fabiani, G., Galaris, E., Russo, L. & Siettos, C. Parsimonious physics-informed random projection neural networks for initial value problems of odes and index-1 daes. *Chaos: An Interdisciplinary Journal of Nonlinear Science* **33** (2023). DOI 10.1063/5.0135903.

[16] Liu, P., Siettos, C., Gear, C. W. & Kevrekidis, I. Equation-free model reduction in agent-based computations: Coarse-grained bifurcation and variable-free rare event analysis. *Mathematical Modelling of Natural Phenomena* **10**, 71–90 (2015). DOI 10.1051/mmnp/201510307.

[17] Frewen, T. A., Hummer, G. & Kevrekidis, I. G. Exploration of effective potential landscapes using coarse reverse integration. *The Journal of*

chemical physics **131**, 10B603 (2009). DOI 10.1063/1.3207882.

[18] Bello-Rivas, J. M. & Elber, R. Simulations of thermodynamics and kinetics on rough energy landscapes with milestoning. *Journal of Computational Chemistry* **37**, 602–613 (2015). DOI 10.1002/jcc.24039.

[19] Patsatzis, D. G., Russo, L., Kevrekidis, I. G. & Siettos, C. Data-driven control of agent-based models: An equation/variable-free machine learning approach. *Journal of Computational Physics* **478**, 111953 (2023). DOI 10.1016/j.jcp.2023.111953.

[20] Reppas, A., De Decker, Y. & Siettos, C. On the efficiency of the equation-free closure of statistical moments: dynamical properties of a stochastic epidemic model on erdős–rényi networks. *Journal of Statistical Mechanics: Theory and Experiment* **2012**, P08020 (2012). DOI 10.1088/1742-5468/2012/08/P08020.
